# Supplementary material for: Peering inside the black box by learning the relevance of many-body functions in neural network potentials
Source: Nat Commun. 2025 Nov 10;16:9898. doi: 10.1038/s41467-025-65863-0 (PMC12603069; doi:10.1038/s41467-025-65863-0)
Supplement: Supplementary file 1 — Supplementary Information [file 41467_2025_65863_MOESM1_ESM.pdf]

Peering inside the Black Box by Learning the Relevance of Many-Body Functions in Neural Network Potentials

Supplementary Information

Klara Bonneau[1†], Jonas Lederer[2,3†], Clark Templeton[1*†], David Rosenberger[1,4], Lorenzo Giambagli[1], Klaus-Robert Müller[2,3,5,6*], Cecilia Clementi[1,7,8*]

[1]Department of Physics, Freie Universität Berlin, Arnimallee 12, 14195, Berlin, Germany.
[2]Machine Learning Group, Technische Universität Berlin, Marchstr. 23, 10587, Berlin, Germany.
[3]BIFOLD - Berlin Institute for the Foundations of Learning and Data, Germany.
[4]BAM Federal Institute for Materials Research and Testing, Unter den Eichen 87, 12205, Berlin, Germany.
[5]Department of Artificial Intelligence, Korea University, Korea University, Seoul, 136-713, South-Korea.
[6]Max Planck Institute for Informatics, 66123, Saarbrücken, Germany.
[7]Center for Theoretical Biological Physics, Rice University, Houston, 77005, TX, USA.
[8]Department of Chemistry, Rice University, Houston, 77005, TX, USA.

*Corresponding author(s). E-mail(s): clarktemple03@gmail.com; klaus-robert.mueller@tu-berlin.de; cecilia.clementi@fu-berlin.de;
†These authors contributed equally to this work.

Contents

S1 Simulation Details **S3**

S1.1 All-Atom Simulations S3

S1.2 CG simulations S3

S2 Neural Network Training **S3**

S3 Comparison with Other Models **S3**

S3.1 Water & Methane S3

S4 Many-Body Decomposition **S6**

S5 Additional Analysis **S10**

S5.1 Individual walk relevances S10

S5.2 Details on 3-body contributions S11

S5.3 Details on the NTL9 analysis S15

S6 Relevance Propagation Procedure of GNN-LRP **S15**

S6.1 Different $\mathbf{x}^*$, Different Rules S18

S6.2 Relevance as Gradient Computation S23

S1 Simulation Details

S1.1 All-Atom Simulations

q_C = -0.36e	q_H = 0.09e
$\epsilon_C = 0.326\ \frac{kJ}{mol}$	$\epsilon_H = 0.100\ \frac{kJ}{mol}$
$\sigma_C = 0.339$ nm	$\sigma_H = 0.239$ nm
$k_{bond} = 269450\ \frac{kJ}{mol \cdot nm^2}$	$r_0 = 0.111$ nm
$k_{angle} = 297\ \frac{kJ}{mol \cdot rad^2}$	$\theta_0 = 1.89$ rad

Table S1: Methane Parameters for simulation in the AA system. Parameters are given for partial charges of Carbon and Hydrogen, LJ interactions, and bond & angle parameters.

S1.2 CG simulations

	Water & Methane	NTL9
Integrator	Langevin	Langevin
Friction	1 ps^{-1}	1 ps^{-1}
Timestep	5 fs	2 fs
Temperature	300K	350K
Number of steps	40000	4×10^6
Masses	Atomistic	Atomistic

Table S2: Parameters for simulation of the CG models of the different systems.

S2 Neural Network Training

	Water & Methane PaiNN	Water & Methane SO3Net	NTL9 PaiNN
RBF type	Gaussian	Gaussian	Gaussian
RBFs	15	20	20
Atom. features	128	128	128
Int. blocks	3	3	3
Lmax	1	2	1
Cutoff	7.5Å	5Å	10Å
Activation	SiLU	SiLU	Tanh

Table S3: Hyperparameters for the different models

S3 Comparison with Other Models

S3.1 Water & Methane

As a comparison to the PaiNN and SO3Net models shown in the main text, Fig. S1 shows the performance of the Inverse Monte Carlo (IMC) method and a SchNet [1] model on the same systems.

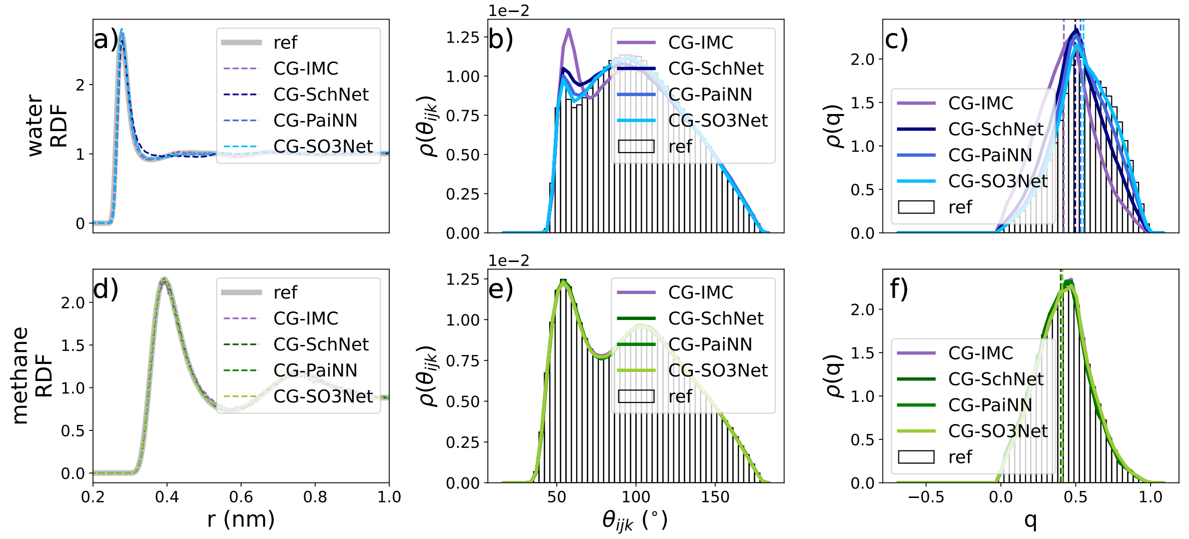

Fig. S1: Performance of various coarse-grained models on water (top) and methane (bottom). In each row, the left plot shows the performance of the models on reproducing the radial distribution function (RDF), the middle plot shows the local angle distribution ($\rho(\theta_{ijk})$) and the right plot shows the tetrahedral order parameter ($\rho(q)$) as defined in Eq. (1). The dashed lines in panels c) and f) show the mean of the corresponding tetrahedral order parameter distribution.

IMC allows one to reconstruct pair potentials from known radial distribution functions (RDFs). This method illustrates the results that can be achieved on these systems if the CG models allow only 2-body interactions.

SchNet can be considered as the predecessor of PaiNN, as it is a message-passing architecture with only invariant ($l_{\text{max}} = 0$) features. It was trained on the atomistic reference data for water and methane with the same procedure as PaiNN and SO3Net. The hyperparameters for the shown SchNet water model are 64 Gaussian RBFs, 256 atomistic features, 6 interaction blocks, 10 Å cutoff, Tanh activation, and for the SchNet methane model 16 PhysNet-style RBFs, 128 atomistic features, 3 interaction blocks, 5 Å cutoff and Tanh activation.

Panels a and d of Fig. S1 show the RDFs obtained with all methods. IMC recovers the RDFs both for water and methane, unsurprising since the RDF was the optimization target of the method. Perhaps more interestingly, while for methane all models perform well, SchNet is not capable of fully recovering the RDF of water, whereas architectures incorporating equivariant features succeed in that attempt. We explain this by the fact that, to compensate for the lack of equivariant features, SchNet may need a much larger cutoff than equivariant architectures to recover directional information crucial to the modelling of CG water. In our experiments, even with a 10 Å cutoff and six interaction blocks, SchNet cannot recover the RDF fully.

Panels b and e of Fig. S1 show the local angle distribution of the different CG models compared to the reference atomistic one. Local angle distributions are computed as the distribution of angles for triplets of beads located inside a cutoff [2, 3] set here to the end of the first solvation shell (3.5Å for

water and 5.6Å for methane), i.e. for all triplets of beads i, j and k (considering all permutations), if $r_{ij} < r_{cut}$ and $r_{jk} < r_{cut}$, then θ_{ijk} is counted in the distribution. The angle distribution for methane in panel e is very similar to the one of a Lennard-Jones fluid with preference for icosahedral packing corresponding to the sharp peak around 63° and the broader peak around 116°. In contrast, water (panel b) has a much stronger peak around 109.5° corresponding to the tetrahedral arrangement and a smaller peak around 50-60° corresponding to interstitial water molecules inside a tetrahedral arrangement. One can see that while for methane all models recover the local angle distribution correctly, for water only the models including many-body terms capture the right overall shape and the inclusion of equivariant features makes the prediction quantitative.

To highlight the importance of the many-body terms further, panels c and f of Fig. S1 show the distribution of the orientational tetrahedral order, q, of the different CG models compared to the corresponding distribution in the reference atomistic model. The orientational order parameter is defined as

$$q = 1 - \frac{3}{8} \sum_{j=1}^{3} \sum_{k=j+1}^{4} \left(cos\, \theta_{jk} + \frac{1}{3} \right)^2 \tag{1}$$

where θ_{jk} is the angle formed by a given bead and its two nearest neighbors j and k [4, 5]. The value of q can vary between -3 and 1 for an individual molecule and the average value for a collection of molecules ranges between 0 and 1 where 0 corresponds to an ideal gas structural arrangement, and 1 corresponds to a perfectly packed tetrahedron [5, 6]. Dashed vertical lines in panels c and f of Fig. S1 show the mean $\langle q \rangle$ value.

On panel f of Fig. S1, one can see that the q distribution for methane is very similar to the one of a Lennard-Jones system [5], driven by pairwise interactions with little to no many-body effects. In contrast, panel c shows that the q distribution for water has a more pyramid-like structure and displays a shoulder corresponding to transient arrangements shown to be stronger at low temperatures [5]. One can see that only the equivariant architectures PaiNN and SO3Net capture this behavior correctly, SchNet fails at reproducing the shoulder and IMC predicts a Lennard-Jones-like distribution.

These results show that for a complex system like bulk water, even if simple methods are correctly capturing the 2-body effects, higher-order features are needed to recover many-body observables. Furthermore, the results emphasize that equivariant features of increasing rotation order appear to help in learning accurate multi-body interactions. In principle, for a sufficiently large amount of data and increasingly large cutoff radii, models based on invariant features should have a complete representation of the system, allowing to accurately learn interactions. However, extensively large cutoff radii slow down model inference and cause information over-squashing [7]. This constitutes a clear advantage of models that employ features of higher rotation order.

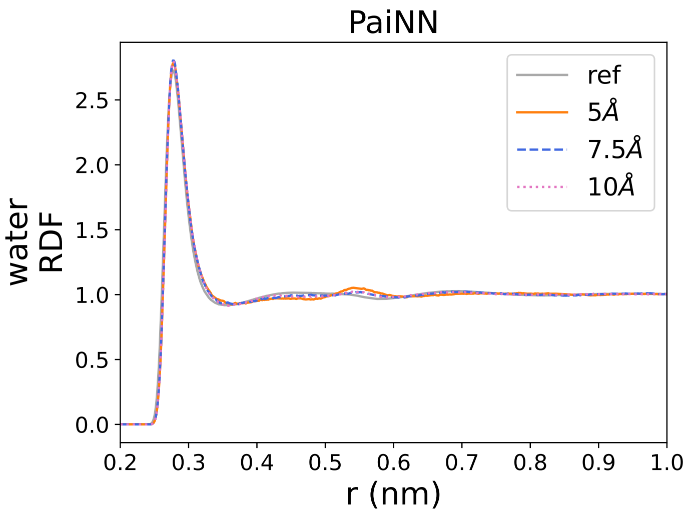

Fig. S2: Performance of PaiNN water models with different cutoff radii. The radial distribution function (RDF) associated with different PaiNN models is shown in comparison to the all-atom reference RDF in grey. The agreement with the reference increases with increasing cutoff radius. In particular, PaiNN with a cutoff radius of 5 Å, does not reproduce the RDF well, while the improvement from 7.5 Å to 10 Å is negligible. Hence, we pick the PaiNN model with cutoff 7.5 Å for the entire analysis shown in the main text.

Figure S2 shows the performance of three PaiNN models with different network cutoffs. The model with a cutoff of 5 Å does not reproduce the RDF correctly. Increasing the cutoff to 7.5 Å corrects for this. Increasing the cutoff even further to 10 Å does not bring any substantial improvement and makes the relevance computation prohibitively expensive (by increasing drastically the total number of walks in the graph), which is why the 7.5 Å cutoff model was chosen for the analysis. For the SO3Net model shown in the main text, a 5 Å cutoff is enough to recover the structural features of the atomistic model.

S4 Many-Body Decomposition

This section aims at highlighting the difference between the LRP approach used in this work and the commonly employed, physically inspired many-body decomposition framework.

It has been shown that the potential energy of a system of N interacting molecules can be formally written as a sum of n-body-order terms, with $n \in 1, ..., N$, giving rise to a many-body expansion of the energy [8]:

$$\begin{aligned} V_N(x_1, x_2, ..., x_N) = &\sum_i V^{1B}(x_i) + \sum_{i<j} V^{2B}(x_i, x_j) + \\ &\sum_{i<j<k} V^{3B}(x_i, x_j, x_k) + ... + V^{NB}(x_1, ..., x_N) \end{aligned} \tag{2}$$

with x_i denoting the coordinates of the atoms in molecule i. V^{1B} corresponds to the deformation energy of an individual molecule and the terms in the sums V^{nB} can then be defined recursively:

$$\begin{aligned} V^{nB}(x_1, x_2, ..., x_n) =& V_n(x_1, x_2, ..., x_n) - \sum_i V^{1B}(x_i) - \sum_{i<j} V^{2B}(x_i, x_j) \\ & - ... - \sum_{i_1<i_2<...<i_{n-1}} V^{(n-1)B}(x_{i_1}, ..., x_{i_{n-1}}) \end{aligned} \tag{3}$$

where $V_n(x_1, x_2, ..., x_n)$ is the model energy for molecules 1 to n.

As one can see, the "n-body contributions" computed by GNN-LRP are not the same as the n-body energy terms of the many-body decomposition. Indeed, GNN-LRP is decomposing the total energy of a full frame into contributions for different walks inside the graph, taking also into account the surroundings of each subset of nodes included in a walk. In contrast, the n-body energy from the many-body decomposition framework corresponds to the energy necessary to form an isolated n-mer of beads/molecules from its sub-elements (monomers, dimers, etc), thus ignoring the effect of the neighboring molecules in a bulk environment. On the more practical side, the computation of the n-body energy in the multi-body decomposition requires computing the network energy prediction for isolated dimers and trimers, configurations that are very far outside of the training configurations containing bulk frames with several hundred beads. We can thus expect an accuracy loss due to the strong network extrapolation.

Following other studies [9, 10, 11], we computed the n-body energies for our water and methane models with n ranging up to 3. The 1-body energy was defined as the energy of an isolated water/methane bead and computed the 2-body and 3-body energies according to Eq. 3.

Concretely, for a dimer composed of beads i and j we computed

$$V^{2B}(x_i, x_j) = E(x_i, x_j) - (E(x_i) + E(x_j)) \tag{4}$$

where $E(x_i, x_j)$ is the model energy predicted for the dimer (i, j), and $E(x_i)$ is the model energy for bead i (that is the same for bead j since the model is invariant with respect to translation).

For a trimer composed of beads i, j and k we computed

$$\begin{aligned} V^{3B}(x_i, x_j, x_k) =& E(x_i, x_j, x_k) - \sum_{dimers} V^{2B}(dimer) - \sum_{monomers} V^{1B}(monomer) \\ &= E(x_i, x_j, x_k) - (E(x_i, x_j) + E(x_i, x_k) + E(x_j, x_k)) \\ &+ E(x_i) + E(x_j) + E(x_k) \end{aligned} \tag{5}$$

where $E(x_i, x_j, x_k)$ is again the model energy prediction for the trimer alone.

We performed this computation for all dimers and trimers present in the interpreted frames from Section 2.1 and plotted the results against the same variables as our relevance scores (i.e., distance for the 2-body energies and largest angle and opposite edge length for the 3-body energies). The results are presented in Fig. S3 for the 2-body energies and Fig. S4 for the 3-body energies.

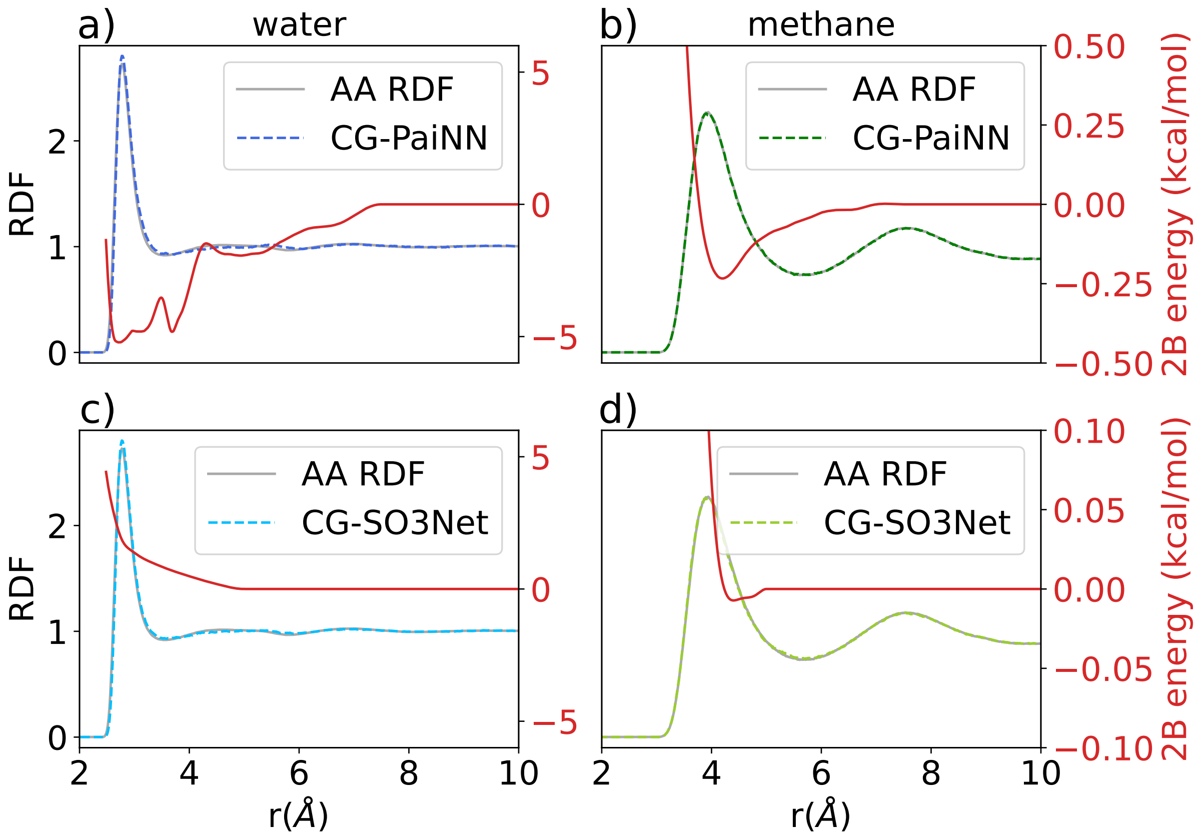

Fig. S3: 2-body energies predicted by the different models presented in Section 2.2 as a function of the distance between the beads. The 2-body energies are compared to the radial distribution functions (RDF) of the atomistic (AA) and coarse-grained (CG) simulations.

It is interesting to note that unlike our relevance score, a bead pair (i, j) only has one 2-body energy value $V^{2B}(x_i, x_j)$ associated, whereas LRP considers the different relevances of this bead pair (e.g., $R_{i,j,i,j}$, or $R_{i,i,i,j}$, etc.) corresponding to the multiple 2-body walks visiting these two nodes. The same is true for a triplet (i, j, k), which only has one associated 3-body energy value $V^{3B}(x_i, x_j, x_k)$. Since the energy of a dimer is entirely determined by the distance between the two beads in the translation- and rotation-invariant models presented here, Fig. S3 shows a simple plot instead of a histogram with mean values of each bin in solid lines and standard deviations as shaded regions.

Fig. S3 shows that the 2-body energy terms from many-body decomposition give the same results as GNN-LRP only asymptotically: at low distances, the energy increases and at distances above the network cutoff the energy becomes zero. The difference with the GNN-LRP prediction is clear on these plots, as the water SO3Net model (Fig. S3 c) only shows repulsive 2-body interactions and the water

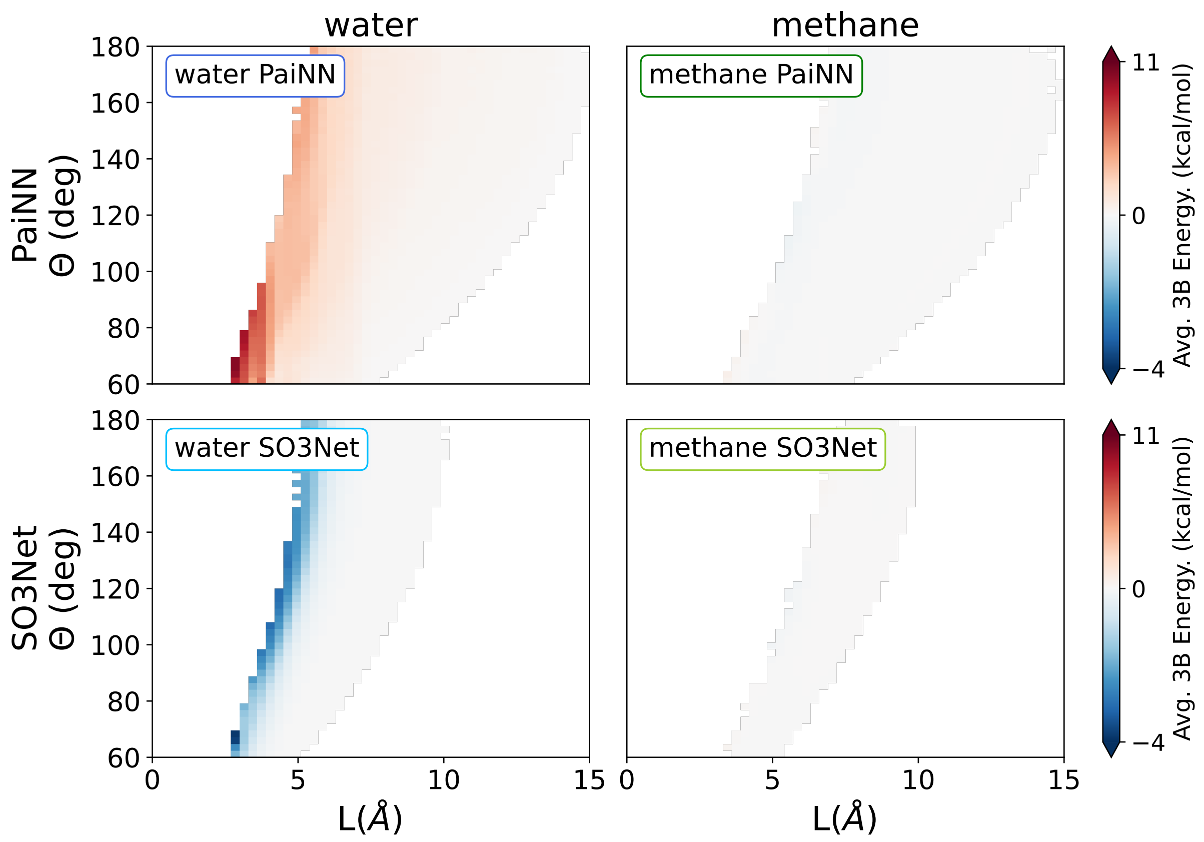

Fig. S4: Average 3-body energies predicted by the different models presented in Section 2.2 as a function of the largest angle in the triangle (θ) and length of the opposite edge (L). Angle and distance ranges are divided in 50 bins each, and colors correspond to average values of the 3-body relevance for all triplets whose largest angle and opposed edge length correspond to the bin.

PaiNN model (Fig. S3 a) shows a very rugged energy prediction. This ruggedness is likely due to the fact that the network prediction is done on an isolated pair of beads constituting a water dimer, which is very different from any frame in the training data distribution, containing 256 water beads.

Supplementary Fig. S4 shows how the 3-body energy as obtained via many-body decomposition is different from LRP (see Fig. 3). One can see that both methods predict 3-body terms for methane that are negligible compared to the ones predicted for water, no matter the architecture. Interestingly, it seems through many-body decomposition that the two architectures PaiNN and SO3Net learn opposite types of 3-body interactions for water, one mostly repulsive and the other one mostly attractive, where LRP showed that both architectures were learning the same overall features. Likely this difference is again due to the fact that the 3-body energy obtained through many-body decomposition required evaluating the network on a single isolated water trimer, which is very different from the training data distribution containing only bulk frames.

While taking into account the effect of the surroundings in the energy decomposition, GNN-LRP also does not amount to a sensitivity analysis, which only focuses on the effect of local changes in the input features on the output, as explained in the introduction of the main text.

S5 Additional Analysis

S5.1 Individual walk relevances

In this work we analyze 2-body and 3-body relevance contributions for water, methane and NTL9. The n-body contributions are obtained by aggregating the relevance scores of particular walks (cf. Section 4.3 in the main text). While this postprocessing provides physically interpretable quantities, it also reduces the information we can gather from GNN-LRP. Hence, here we analyze the relevance attributions directly provided by GNN-LRP for 2-body walks (walks that only involve a single pair of beads).

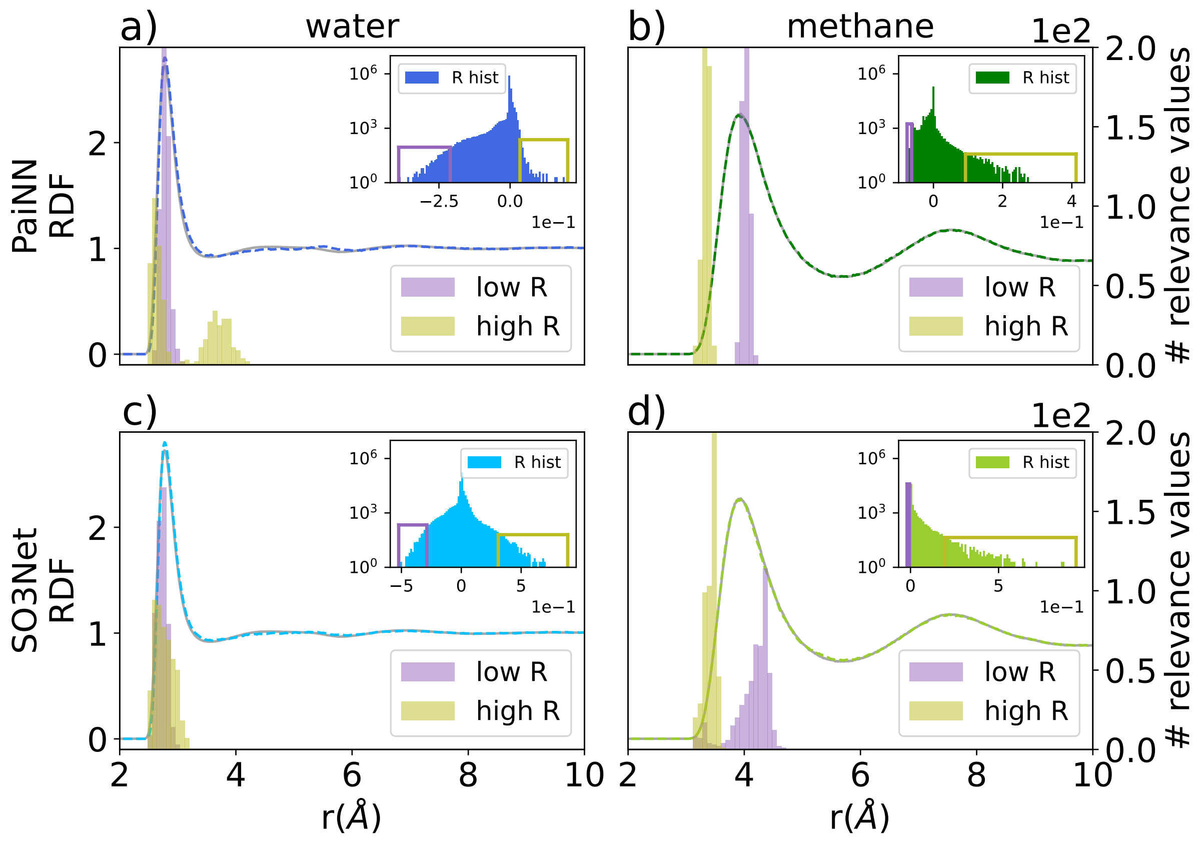

Fig. S5: Highest and lowest 2-body walk relevance attributions as a function of the distance. For each model and system, the distribution in distance of the 500 highest and lowest relevance values is plotted alongside the radial distribution functions (RDF) of atomistic (AA, full grey line) and coarse-grained (CG, dashed colored lines) simulations. As an inset is shown the entire distribution of 2-body relevance attributions with rectangles delimiting the region where the 500 highest and lowest values are located.

Here, we consider 2-body walks for methane and water. Figure S5 shows the pairwise distances corresponding to those 2-body walks with lowest and highest relevance attributions. Since low/high relevance indicates strong attraction/repulsion, the associated distances correspond to the most stabilizing or destabilizing interactions flagged by the network. For all systems and models, the lowest relevance values correspond to distances in the first solvation shell, deemed stabilizing by the models. Destabilizing interactions (high positive values) are located at distances too short to be in the first solvation shell

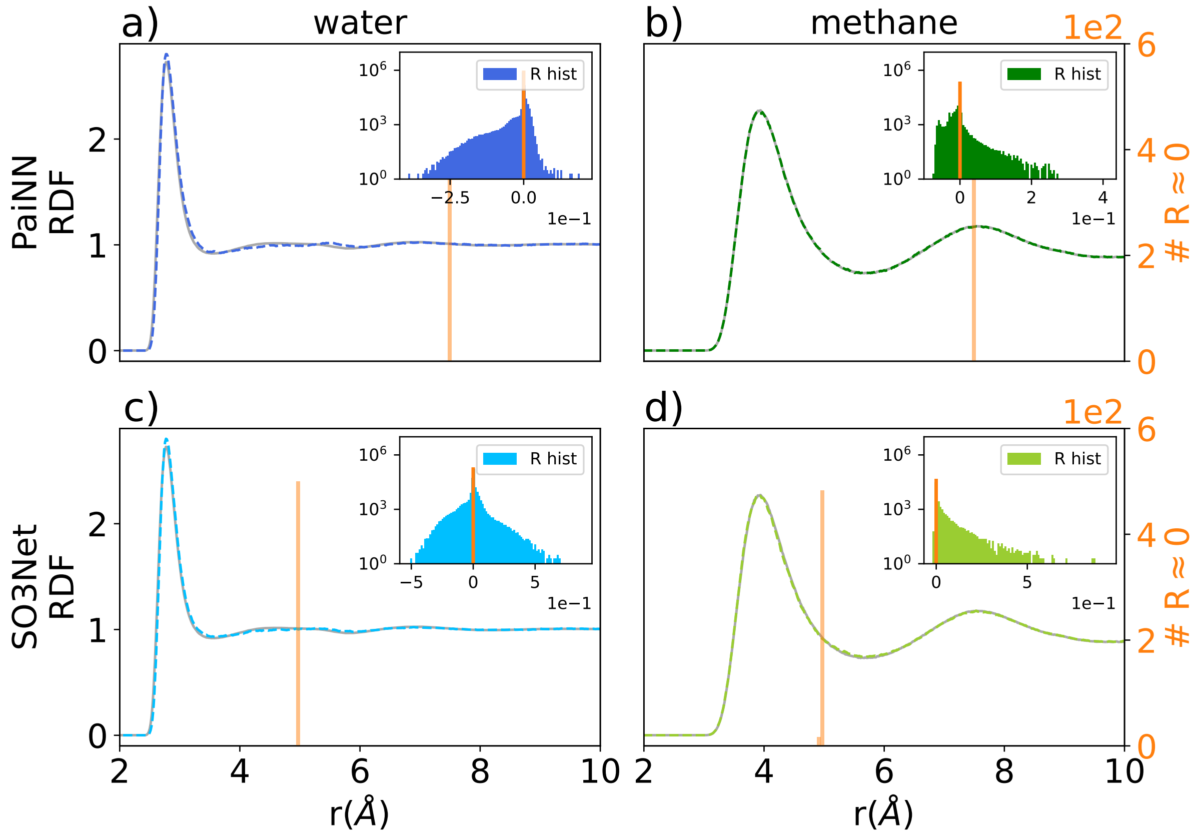

Fig. S6: Zero 2-body relevance scores as a function of the distance. For each model and system, the distribution in distance of the null relevance values is plotted alongside the radial distribution functions (RDF) of atomistic (AA, full grey line) and coarse-grained (CG, dashed colored lines) simulations. As an inset is shown the entire distribution of 2-body relevance attributions.

(onset of the RDF) and for PaiNN water (panel a) also for distances between the first and the second solvation shell. Figure S6 shows the distances that have a zero relevance, i.e. that do not contribute to the network prediction. Reassuringly, one can see that these distances correspond exactly to the network cutoff in each case.

S5.2 Details on 3-body contributions

Figure S7 shows the average 3-body relevance contributions for all four models alongside the distribution of angles and edge lengths for the training datasets and the CG simulations with the trained NNPs. For the atomistic datasets and CG simulations, a strided portion of the available frames was taken (250 frames in total for each plot), for which all possible walks given a specified network cutoff were computed. All triplets involved in 3-body walks were then extracted and the largest angle and opposed edge length were computed for the triangle formed by the triplet. Two-dimensional histograms are then plotted for both the atomistic distribution and the CG simulations. As could be already inferred from Fig S1, all CG models reproduce the atomistic distribution correctly. Interestingly, the distribution of average 3-body relevance contributions does not correlate with the angle/distance distributions, showing

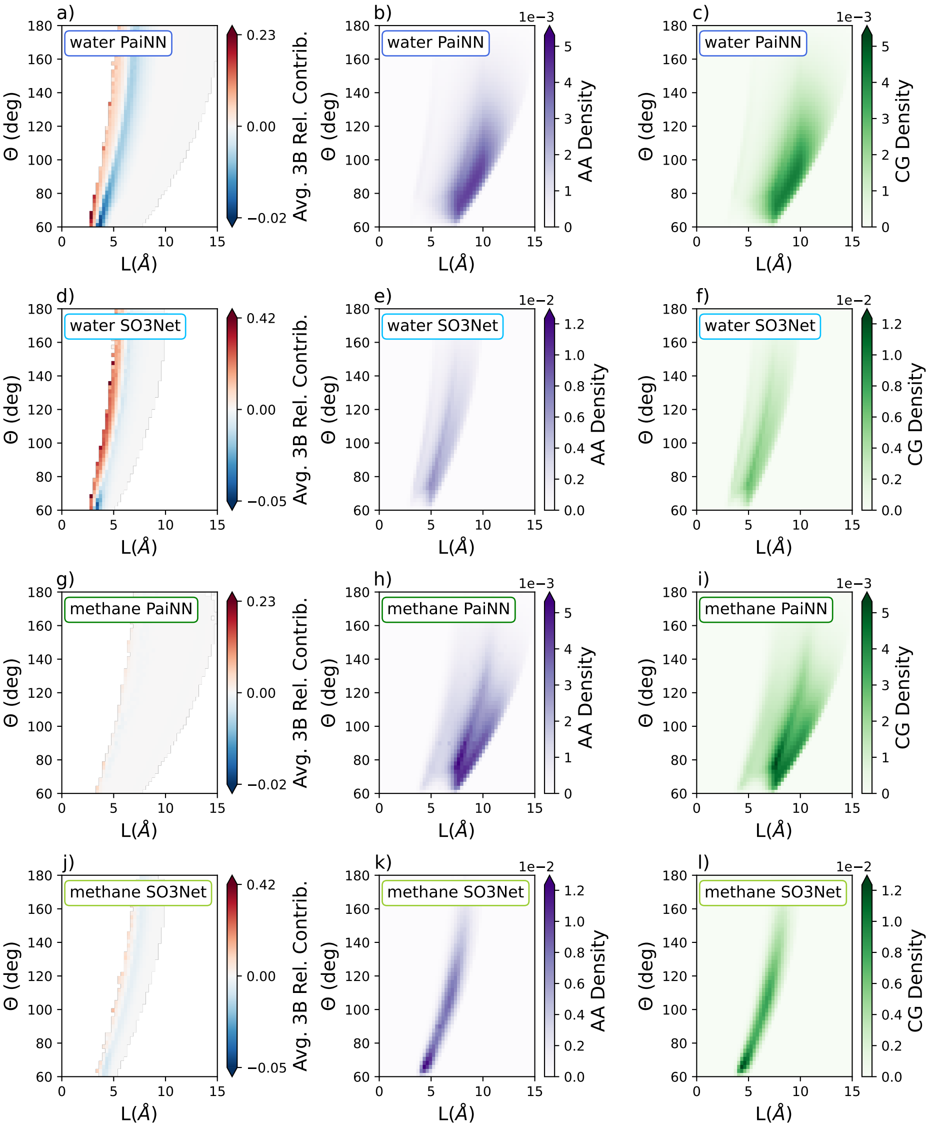

Fig. S7: 3-body relevance contributions (a, d, g and j) compared with distributions of angles (θ) and edge lengths (L) for the atomistic (AA) training datasets (b, e, h and k) and the distributions produced by the coarse-grained (CG) simulations using the trained Neural Netowrk Potentials (NNPs) (c, f, i and l). Note that b and h show distributions from the same atomistic training dataset for water, the difference occurs only because the different cutoffs for PaiNN and SO3Net produce different sets of triplets with associated relevance. The same point goes for e and k that both stem from the same methane atomistic simulations.

that the 3-body interactions learned by the NNPs are not trivial. Note that the atomistic distributions for each system differ between models only because of the different network cutoff used. Figs. S7b and d (the same point goes also for e and k) show distributions from the same frames, but the different network cutoffs between PaiNN and SO3Net result in a different set of triplets involved in 3-body walks such that the distributions look different.

As the two features chosen to describe a triplet of beads (largest angle and length of opposite edge) do not fully describe a triangle, in the following we illustrate how the relevance varies as a function of three features fully describing the triangle formed by the involved triplet: largest angle and lengths of the

two adjacent edges. To illustrate the results we proceed as follows: For each triplet of beads we compute the associated relevance according to Eq. 11. We then compute the largest angle (θ) and length of the two adjacent edges ($L1$ and $L2$, $L1$ being the shortest one) for each triplet and obtain a 3-dimensional histogram where each voxel corresponds to a value of θ, $L1$ and $L2$. In each voxel, we average values of relevance contributions just as was done in Fig. 3 for the 2-dimensional histogram. We can then plot a scatter plot in 3 dimensions where each voxel has the color corresponding to the average 3-body contributions value. An example of such a 3-dimensional plot for the PaiNN water model is shown in the left panel of Supplementary Fig. S8. In order to enable better comparison between the models, these 3-dimensional plots are then projected into slices in Supplementary Figs. S9 and S10. Supplementary Fig. S8 illustrates on one example how the slices in the following two figures were obtained. Because of the geometrical constraints in a water or methane box, all 3-dimensional distributions have the shape of a triangular prism with one edge located at minimum $L1$ and $L2$ fixed by the location of the initial increase in the RDF (($L1 = 2.55$ Å, $L2 = 2.55$ Å) for water and ($L1 = 3.15$ Å, $L2 = 3.45$ Å) for methane). Each slice in Supplementary Figs. S9 and S10 corresponds to a slice taken with a different inclination of a plane around a hinge on the axis corresponding to minimum $L1$ and $L2$ (see first row in Supplementary Fig. S8. The points within each slice are illustrated on the second row of Supplementary Fig. S8. These points are then projected onto the ($L2$, θ) plane as illustrated by the eye. Each projection corresponding to a different inclination of the slice (α) is then illustrated in a different panel in Figs. S9 and S10.

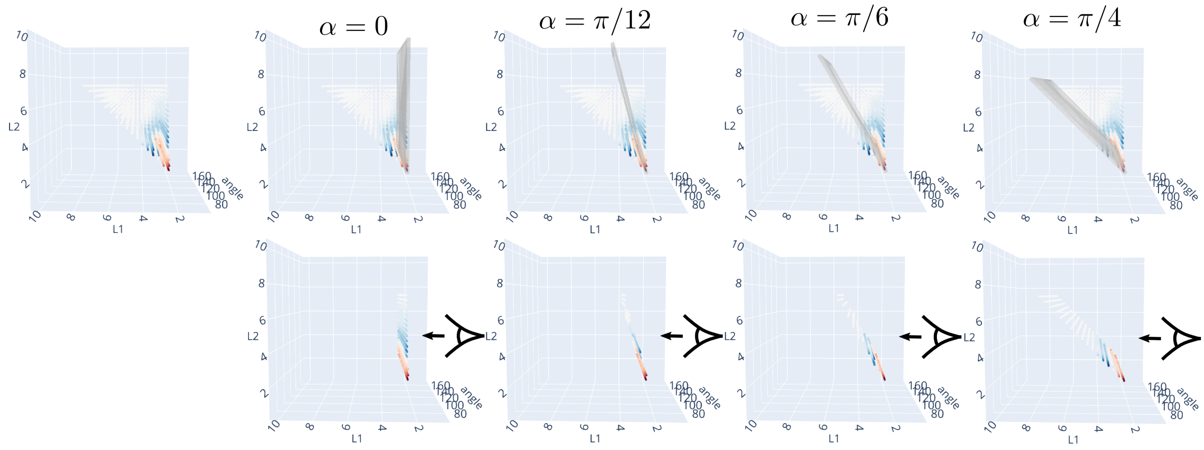

Fig. S8: Illustrative explanation of how the waterfall plots in Supplementary Figs. S9 and S10 were obtained.

Fig. S9 shows the 2-dimensional projection of the previously described slices of the 3-dimensional distribution of 3-body relevance contributions. Interestingly, while on Fig. 3 it appeared that the relevance for methane was always very close to zero, the three-dimensional plot shows that for specific bins located exactly at minimum $L1$ (smallest edge in the triangle at 3.15 Å), the interpretation of the

PaiNN methane model shows some non-negligible stabilizing relevance contributions, that are averaged out when reporting as a function only of the length of the largest edge.

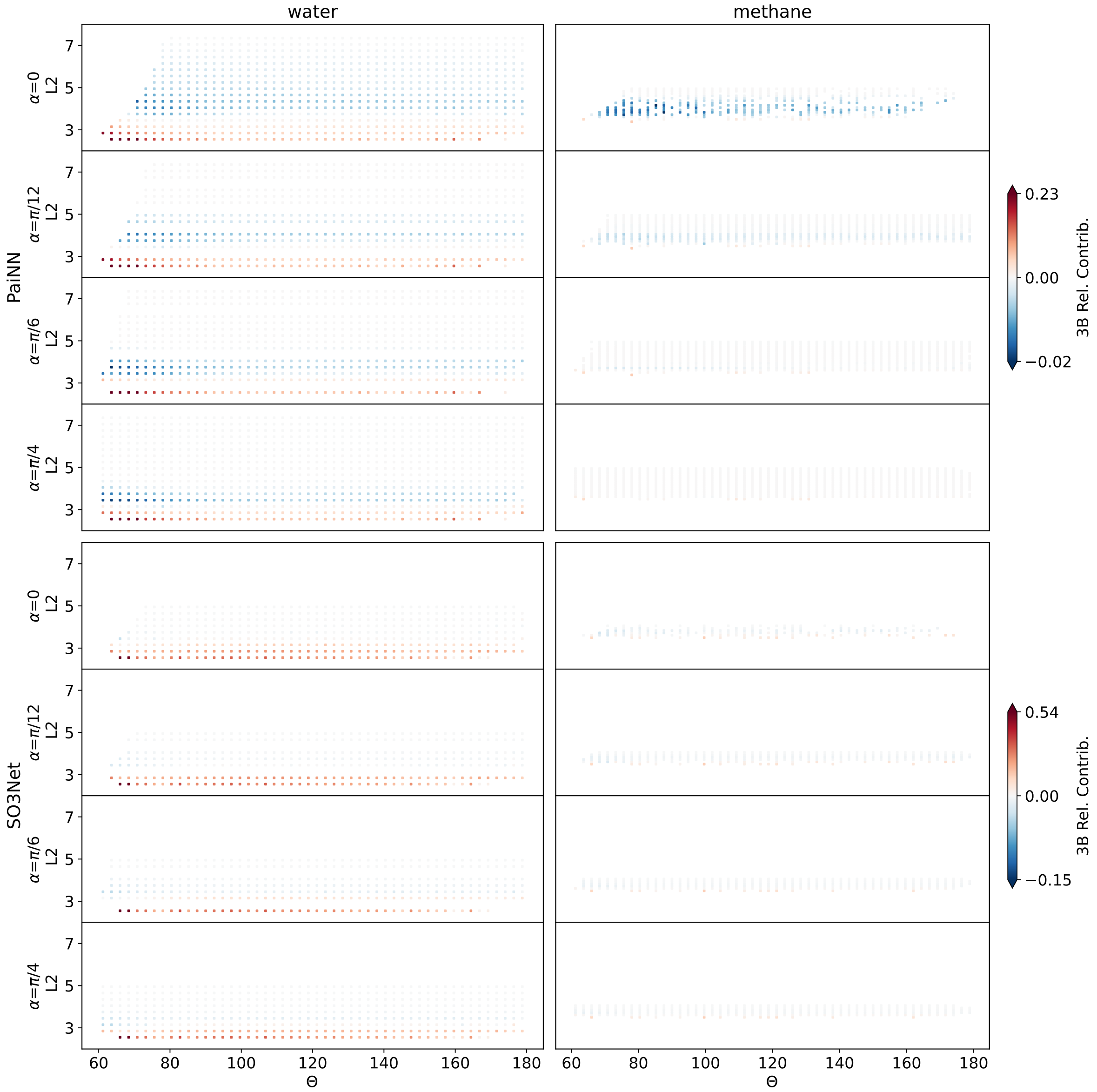

Fig. S9: Three-dimensional distribution of 3-body relevance contributions as a function of the largest angle in the triangle (θ) and the lengths of the two adjacent edges ($L1$ and $L2$). The four slices are obtained with a rotating slice at different angles α, illustrated in Supplementary Fig. S8, and projected onto the $L2$ dimension. The hinge for the rotating slice for water is located at ($L1 = 2.55$ Å, $L2 = 2.55$ Å) and for methane at ($L1 = 3.15$ Å, $L2 = 3.45$ Å)

This minimum $L1$ value where all the stabilizing 3-body contributions for methane are obtained is located before the onset of the RDF, where the density is close to zero, the model should thus not learn to stabilize these distances. In order to explain this, we looked at the number of interpreted triplets that fall into each bin, illustrated in Fig. S10. As one can see, the number of data points for this $\alpha = 0$ slice in the methane models is very small (1 to 10 data points only), meaning that the prediction of

the 3-body terms in these bins is probably not good, compared to the other bins in the water models that have on the order of several thousand points. This apparent stabilization of the shorter distances by the 3-body contributions of the PaiNN methane model does not show in MD simulations, indeed, the 3-body contributions are one order of magnitude smaller than the 2-body contributions that handle the repulsive interactions at shorter distance. This highlights an interesting point: despite having a seemingly well-performing model in MD simulations, the analysis of the model with GNN-LRP shows where the PaiNN methane model falls short and suggests some place for improvement. For example, one could add some conformations with more low distances (and high repulsive forces) obtained through adaptive sampling strategies to the training set to attempt at improving the model.

S5.3 Details on the NTL9 analysis

Figure S11 shows the distances between residues in the interpreted structures for protein NTL9. Compared to panel a of Fig. 5 in the main text, panel a of Fig. S11 shows that PaiNN is capturing the interaction decay with the distance between residues. The comparison between panel b of Fig. 5 in the main text and panels b and c of Fig. S11 shows that the 2-body interactions learned by the network between residues contain much more information than just pairwise distances, as they report on the stability of secondary structure elements inside the intermediate states. Alongside Fig. S7, this illustrates again that the model learns more than mere structural statistics derived from the training dataset.

S6 Relevance Propagation Procedure of GNN-LRP

In this section we describe how the relevance attribution is obtained using efficient backpropagation under consideration of the respective propagation rules. Furthermore, it is explained in detail how relevance attributions are obtained for PaiNN and SO3Net. We will present the LRP rules showing how different forms of $q_{\alpha\beta}$ in Eq. (6) in the main text, can be obtained. We will be starting from the first-order Taylor expansion (that we recall in the following) and focus on the selection of the expansion point. According to Eq. (4) in the main text, the relevance of neuron β in the last neural network layer may be expressed as

$$R_\beta = \sigma(\mathbf{w}_\beta \mathbf{x} + b) = \Theta[\mathbf{w}_\beta(\mathbf{x} - \mathbf{x}^*)]\mathbf{w}_\beta \cdot (\mathbf{x} - \mathbf{x}^*) = \sum_\alpha R_{\alpha\leftarrow\beta} \ . \tag{6}$$

The equation above implies that the expansion point $\mathbf{x}^*$ belongs to the ReLU hinge $\hbar$ so that the zero-th order of the expansion disappears. However, the scalar condition $\mathbf{x}^* \in \hbar$ when $\dim(\mathbf{x}^*) > 1$ is insufficient to fully determine all the components of $\mathbf{x}^*$. Instead, for each neuron we obtain an individual Taylor expansion with its own root point. Consequently, additional criteria must be introduced to address this

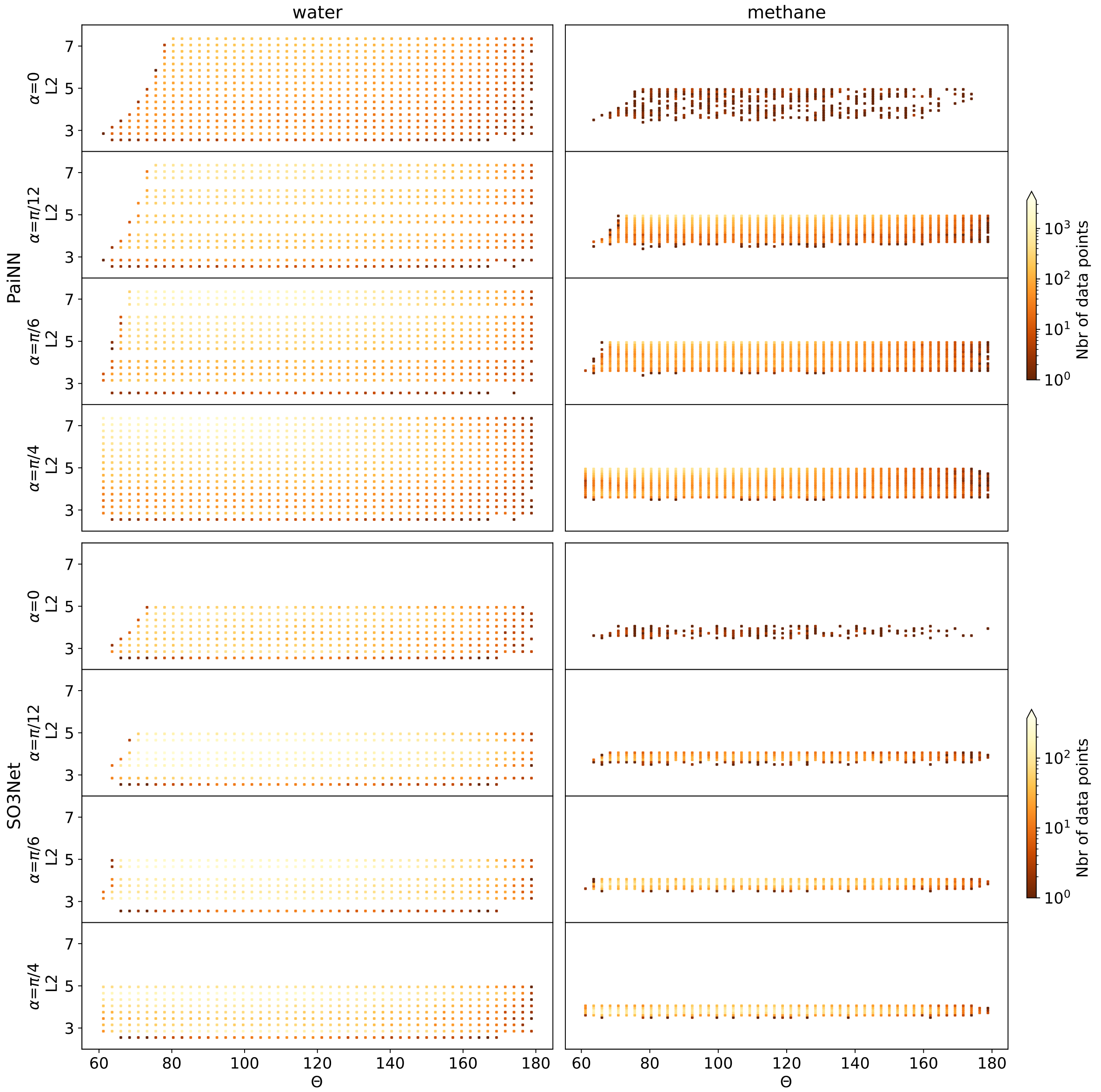

Fig. S10: Three-dimensional distribution of features (largest angle in the triangle (θ) and the lengths of the two adjacent edges($L1$ and $L2$)) in the interpreted triplets. The four slices are obtained with a rotating slice at different angles α, illustrated in Supplementary Fig. S8, and projected onto the $L2$ dimension. The hinge for the rotating slice for water is located at ($L1 = 2.55$ Å, $L2 = 2.55$ Å) and for methane at ($L1 = 3.15$ Å, $L2 = 3.45$ Å)

ambiguity. These criteria should be specifically designed to align with the properties of the network. To ensure a meaningful attribution of relevance to each input component x_α, it is essential to determine whether the point $\mathbf{x}^*$ depends on $\mathbf{x}$ and, if so, specify such dependency.

In order to understand the role of the evaluation point and the interaction with the model, we shall make a digression on the specialization of representation in a neural network layer. It is important to stress that the following explanation uses an intuitive representation of a neural network that is meant to justify the usage of different expansion points $\mathbf{x}^*$. Such explanation, however, can be substituted by

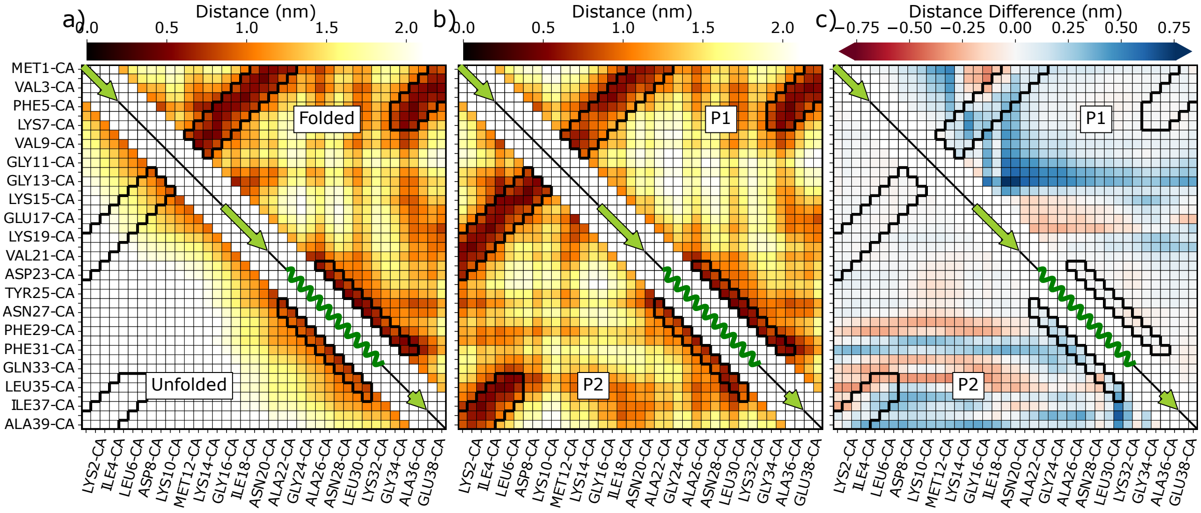

Fig. S11: Mean distances between residues inside the interpreted structures. Panels a) and b) show the mean distances inside the structures from the folded, unfolded, P1 and P2 states and panel c) shows the distance difference between the folded state and the P1/P2 states (i.e. $d_F - d_{P1/P2}$).

the empirical evidence that valid propagation rules can be obtained using different expansion points and that empirically, some work better than others.

In a neural network, each data point undergoes multiple non-linear transformations at every layer. We define the *representation* of a data point as the activation of a specific neuron in response to that input. Collectively, the activations of all neurons in a given layer form the coordinates of a vector that represents the input, capturing specific features related to the neurons' receptive fields, namely, their connections. Let us assume that we have a proper functioning neural network, namely a neural network with good out of sample error (i.e., test error) in a classification scenario. We shall expect the representation at the end to be linearly separable and each class properly attributed. Assume that we have four classes, and that we are in the idealized scenario where the neural network's hidden representations are well separated, namely the class conditioned representation "does not overlap too much" in the vector space. This phenomenon could happen in two distinct ways, each represented in Fig. S12.

- Correlated representations (Fig. S12a): Activations of neurons within or across groups (e.g., Activation Groups 1 and 2) exhibit statistical dependencies. Here, relevance attribution must account for overlapping receptive fields.
- De-correlated representations (Fig. S12b): Activations are disentangled, with minimal overlap between classes or neuron groups and negligible statistical correlation between different groups. Relevance can propagate through more independent pathways.

In correlated regimes (Fig. S12a), a single global expansion point may inadequately capture local feature interactions, leading to blurred attributions. Conversely, in de-correlated regimes (Fig. S12b), neuron-specific expansion points better isolate contributions from independent features. Thus, the optimal $\mathbf{x}^*$ depends on whether the network's latent space prioritizes feature redundancy (correlated) or feature

specialization (de-correlated). Those two different regimes induce two different approaches in the choice of the point $\mathbf{x}^*$. More specifically we shall expect the scalar product between $\mathbf{x}$ and the vector $\mathbf{w}_\alpha$ in equation (6) to be more informative for the case of de-correlated representation S12b, whereas in Fig. S12a we should also expect the scalar product alone to be not enough and therefore some shift in the expansion point should be also employed. Indeed, for the case of de-correlated representations we will assume that an evaluation point independent from $\mathbf{x}$ is still informative enough, as opposed to the case of correlated representations, where a relation $\mathbf{x}^*(\mathbf{x})$ should be introduced.

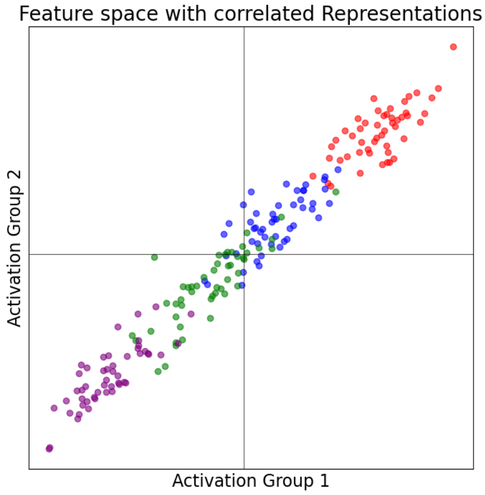

(a) Correlated representations: Activations of neurons within or across groups (e.g., Activation Groups 1 and 2) exhibit statistical dependencies, leading to overlapping feature contributions.

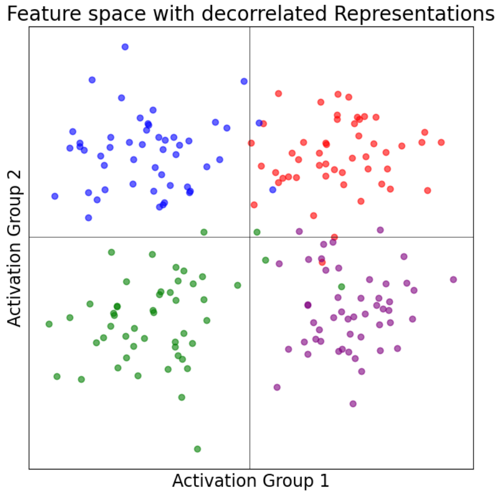

(b) De-correlated representations: Activations are disentangled, with minimal overlap between classes or neuron groups, enabling independent feature contributions.

Fig. S12: Schematics of disentangled but correlated (a) and de-correlated (b) representations. Here, we assume the existence of two families of neurons whose activations define the components of the embedding. Each scenario illustrates distinct feature interaction patterns: correlated representations (left) exhibit overlapping contributions, while de-correlated representations (right) emphasize independent feature contributions. This is an oversimplified, low-dimensional cartoon; the actual situation is much more complex.

S6.1 Different $\mathbf{x}^*$, Different Rules

In this section, we will show how different rules arise. We will not cover all of them as a more detailed list can be found in literature [12]. We, however, present examples and more explicit proofs for the most relevant ones, starting from the simplest and moving towards the more elaborate one, that was actually used in this work.

0-Rule: $\mathbf{x}^* = 0$

The simplest possible rule is the so-called 0-rule, namely $\mathbf{x}^* = \mathbf{0}$ and it will indeed work for ideal situations of de-correlated representations. This assumes no dependence of the expansion point from the

evaluation point, assuming a “context-free” explanation. The latter produces $R_\alpha = \frac{x_\alpha w_{\alpha\beta}}{\sum_\alpha x_\alpha w_{\alpha\beta}} R_\beta$ and is equivalent to a Gradient × Input propagation rule [13].

w^2-Rule: $\mathbf{x}^* =$ nearest point to $\mathbf{x}$ in $\hbar$

The first attempt to insert some context in the explanation of the neural network, is to couple the expansion and evaluation point assuming that the former one is the closest to the latter on $\hbar$. This produces the so-called w^2-rule, that, indeed, selects as expansion point the nearest one to $\mathbf{x}$ that lies in the manifold $\hbar$.

In the following, we will illustrate the calculations for this case in a minimal scenario of one neuron β with 2-dimensional input, so that the impact of the assumption we made will become clear.

The function on neuron β is the ReLU, defined as:

$$f(x_1, x_2, x_3) = \max(x_1 w_1 + x_2 w_2 + b x_3, 0) \tag{7}$$

omitting the index β of $\mathbf{w}$. Moreover, $\mathbf{x}^* \in \hbar$ implies (remembering that we fix $x_3 = 1$)

$$x_1^* w_1 + x_2^* w_2 + b = 0 \tag{8}$$

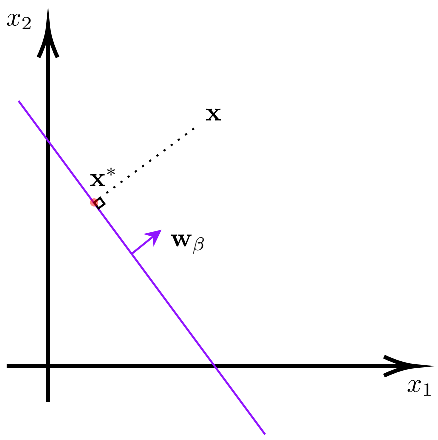

The expansion point $\mathbf{x}^* = (x_1^*, x_2^*, 1)$ being the closest one to the input $\mathbf{x}$ implies $\mathbf{x} - t\mathbf{w} = \mathbf{x}^*$ with $\mathbf{x}^* \cdot \mathbf{w} - b = 0$, therefore (performing the scalar product with $\mathbf{w}$ and substituting $\mathbf{x}^* \cdot \mathbf{w}$):

$$\mathbf{x} \cdot \mathbf{w} - t|\mathbf{w}|^2 + b = 0 \Rightarrow t = \frac{\mathbf{x} \cdot \mathbf{w} + b}{|\mathbf{w}|^2} \tag{9}$$

So the expansion point is:

$$\mathbf{x}^* = \mathbf{x} - \frac{(\mathbf{x} \cdot \mathbf{w}_\beta + b)}{|\mathbf{w}|^2}\mathbf{w} \tag{10}$$

Now we can plug this into (6). In doing so we write explicitly the components, obtaining the value of $R_{\alpha\leftarrow\beta}$ as:

$$R_{\alpha\leftarrow\beta} = \frac{w_{\alpha\beta}(w_{\alpha\beta}\left(\mathbf{x}\cdot\mathbf{w}_\beta + b\right))}{|\mathbf{w}|^2} \tag{11}$$

Assuming multiple neurons, labeled by index β:

$$R_\alpha = \sum_\beta \frac{w_{\alpha\beta}^2}{|\mathbf{w}_\beta|^2}\left(\mathbf{x}\cdot\mathbf{w}_\beta + b\right)\Theta\left[(\mathbf{x}\cdot\mathbf{w}_\beta) + b_\beta\right] \tag{12}$$

Now we consider that $\left(\mathbf{x}\cdot\mathbf{w}_\beta + b\right)\Theta\left[(\mathbf{x}\cdot\mathbf{w}_\beta) + b_\beta\right]$ is equal to the activation of neuron β and therefore, its relevance R_β, and we incorporate the bias as an input connection coming from a neuron of constant activation 1:

$$R_\alpha = \sum_\beta \frac{w_{\alpha\beta}^2}{|\mathbf{w}_\beta|^2} R_\beta \quad \alpha \in \{1, \ldots, N_\kappa + 1\}\ \beta \in \{1, \ldots, N_{\kappa+1}\} \tag{13}$$

This is a relevance propagation rule that can be applied recursively in multilayered cases. The relevance can be back-propagated but only for non-bias neurons, as for $\alpha = 3$ (in this example) we could not back-propagate again as it has no input. We will, reasonably expect that this naive approach will work for cases where $\frac{b}{|\mathbf{w}|^2} \ll 1$.

γ-Rule: interpolation between the 0 and w^2 rules

Further flexibility in the propagation rule can be obtained interpolating between those two regimes, namely strongly context dependent explanations (the w^2-rule) and context free explanation (the 0-rule), considering an expansion point $\mathbf{x}^*$ that depends on a parameter, γ, that lies in the intersection between $\hbar$ and $\ell_{t,\gamma}$ defined as follows:

$$\ell_{t,\gamma} = \mathbf{x} - t(\mathbf{x} + \gamma\mathbf{x}\Theta[\mathbf{w}]) \Rightarrow \ell_{t,\gamma}|_\alpha = x_\alpha - t(x_\alpha + \gamma x_\alpha \Theta[w_\alpha]) \tag{14}$$

This results in a γ-dependent rule that, as we will show, indeed interpolates between the two regimes 0 and w^2 rule. For the sake of clarity we should consider $\ell_{t,\gamma} = \mathbf{x} - t\mathbf{z}_\gamma$ with $\mathbf{z}_\gamma = (\mathbf{x} + \gamma\mathbf{x}\Theta[\mathbf{w}])$, as the position of vector $\mathbf{z}$ will carry interesting information.

To understand the effect of such rule we will use the following schematic that despite being low dimensional still carries insights. Again we consider the 2 dimensional input scenario and a single ReLU neuron.

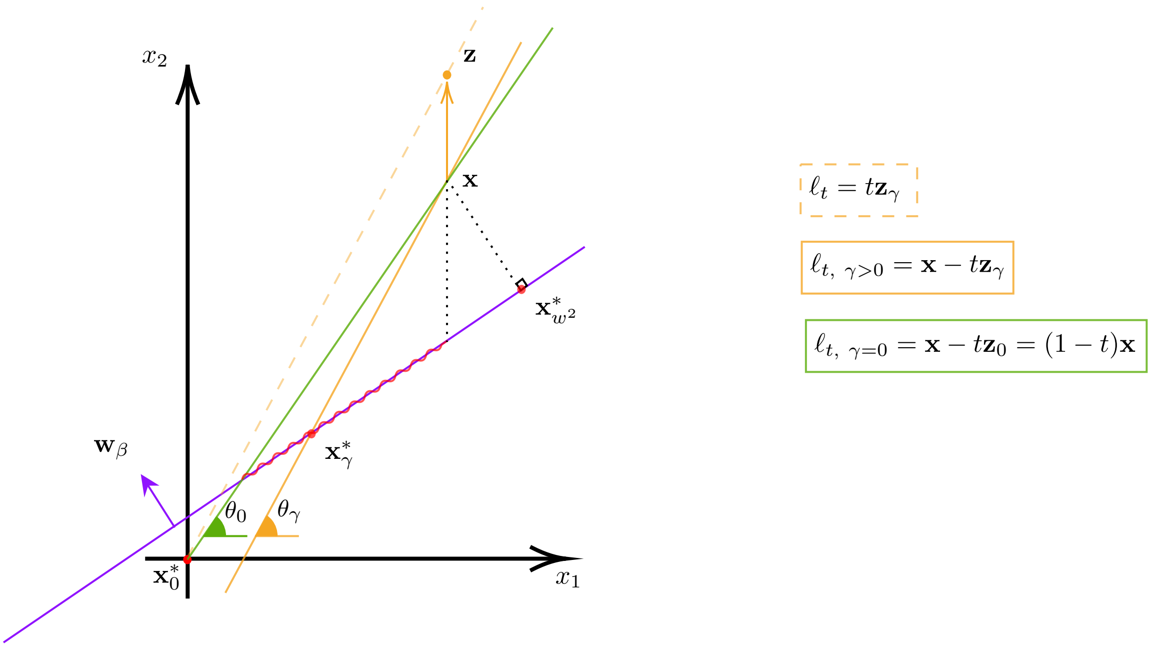

First of all, to find the position of the evaluation point $\mathbf{x}^*_\gamma$ we will construct the line $\ell_t = t\mathbf{z}_\gamma$ in dashed yellow. The position of $\mathbf{z}$ being, in this case where $w_\alpha < 0$ and $w_\beta > 0$, equal to $\mathbf{z}_\gamma = (x_\alpha, (1+\gamma)x_\beta)$ therefore always 'higher' than $\mathbf{x}$. The dashed yellow line in the figure determines the slope of $\ell_{t,\gamma}$ (θ_γ). Therefore, the latter can be drawn by rigidly shifting the dashed yellow line to pass through $\mathbf{x}$. The components ratio of $\mathbf{z}_\gamma$ gives $\tan\theta_\gamma = \dfrac{(1+\gamma)x_\beta}{x_\alpha}$ and $\mathbf{x}^*_\gamma \in \ell_\gamma \cap \hslash$. The extrema can be found considering $\gamma = 0$, the green line, and $\gamma \to \infty$, namely the vertical line passing through $\mathbf{x}$. In this scenario we have that the position of $\mathbf{x}^*_\gamma$ could be in the undulated red region of $\hslash$, indeed interpolating between 0 and the nearest point. Remember that, again, $\frac{b}{|\mathbf{w}_\beta|^2} \ll 1$. Carrying out all the calculation with the imposed expansion point brings to the following relevance propagation rule:

$$R_\alpha = \sum_\beta \frac{x_\alpha \cdot (w_{\alpha\beta} + \gamma w^+_{\alpha\beta})}{\sum_{0,\alpha} x_\alpha \cdot (w_{\alpha\beta} + \gamma w^+_{\alpha\beta})} R_\beta \tag{15}$$

Where $w^+_{\alpha\beta} = \max(w_{\alpha\beta}, 0)$.

This cartoon, despite being simple, grasps the idea behind the γ-rule: higher values of gamma imply higher dependence of the evaluation point with respect to the input making the relevance propagation more 'specific'. This is likely to be more useful when representations inside the network are correlated. On the other hand, small values of γ imply a less input-specific relevance propagation rule: referencing with Equation (6) the scalar product between $\mathbf{w}_\alpha$ and $\mathbf{x}$ is already a good proxy of the activity (relevance) of the neuron; we are indeed closer to the case of de-correlated representations.

Generalized γ-Rule: fine-tuning the effect on negative components of w_β

Now we will show how the generalized gamma rule, used in the paper, connects with the gamma rule

in terms of evaluation points. We will start reporting the full expression of such rule:

$$\begin{aligned} R_\alpha = &\sum_\beta \frac{x^+_\alpha \cdot (w_{\alpha\beta} + \gamma w^+_{\alpha\beta}) + x^-_\alpha \cdot (w_{\alpha\beta} + \gamma w^-_{\alpha\beta})}{\sum_\alpha x^+_\alpha \cdot (w_{\alpha\beta} + \gamma w^+_{\alpha\beta}) + x^-_\alpha \cdot (w_{\alpha\beta} + \gamma w^-_{\alpha\beta})} \cdot I(z_\beta > 0) \cdot R_\beta \\ + &\sum_\beta \frac{x^+_\alpha \cdot (w_{\alpha\beta} + \gamma w^-_{\alpha\beta}) + x^-_\alpha \cdot (w_{\alpha\beta} + \gamma w^+_{\alpha\beta})}{\sum_\alpha x^+_\alpha \cdot (w_{\alpha\beta} + \gamma w^-_{\alpha\beta}) + x^-_\alpha \cdot (w_{\alpha\beta} + \gamma w^+_{\alpha\beta})} \cdot I(z_\beta < 0) \cdot R_\beta \end{aligned} \tag{16}$$

Where $w^-_{\alpha\beta} = \min(w_{\alpha\beta}, 0)$. We will focus on the case $z_\beta > 0$, namely on the subspace of neurons of a given layer with positive activation. The case of negative ones can be constructed with symmetry argument.

The corresponding evaluation point of this rule can be found considering that (S6.1) is the result of two conditions that are acting on two orthogonal subspaces of the input: the one of positive and negative components of $\mathbf{x}$. We shall identify those subspaces as $\mathbb{V}^+$ and $\mathbb{V}^-$ respectively, such that $\mathbb{V} = \mathbb{V}^+ \oplus \mathbb{V}^-$, with $\mathbf{x} \in \mathbb{V}$. The orthogonal projectors that project onto those spaces will be called P^+ and P^- respectively. We will therefore have $\mathbf{x} = \mathbf{x}^+ + \mathbf{x}^- = P^+\mathbf{x} + P^-\mathbf{x}$ with $x^+_\nu >= 0$ and $x^-_\mu < 0$ for all ν, μ in opportune range.

The main idea is to remap the whole rule into γ-rule like evaluation point that are defined on $\mathbb{V}^+$ and $\mathbb{V}^-$. This is done using two ingredients: the ReLU hinge $\hbar$ and an appropriate variation of ℓ_γ. We will start with the latter, considering two curves, one for each subspace. This idea stems from the observation that if $\mathbb{V}^+ = \mathbb{V}$ than the rule becomes the γ-rule and, if $\mathbb{V}^- = \mathbb{V}$ the rule becomes the one considering the following $\hbar$ intersecting curve:

$$\ell^-_{t,\gamma}|_\alpha = x_\alpha - t(x_\alpha + \gamma x_\alpha \Theta[-w_\alpha]) \tag{17}$$

We will therefore consider one curve on each subspace. For clarity, we will refer to the γ-rule generating curve, former $\ell_{t,\gamma}$ as $\ell^+_{t,\gamma}$.

Now we need to rewrite the manifold $\hbar$ projecting it into each subspace $\mathbb{V}^-$ and $\mathbb{V}^+$. Doing so, however, requires a little attention as the manifold $\hbar$ is a global condition on the components and it is not obvious that it could be mapped into analogous conditions on the subspaces $\mathbb{V}^{+,-}$. Luckily such condition involves only linear operators. We can therefore decompose it into two conditions, one for each subspace, exploiting the fact that $\mathbb{I} = P^+ + P^-$.

Lets define $\mathbf{w}^- = P^-\mathbf{w}$ and $\mathbf{w}^+ = P^+\mathbf{w}$. We should remember, however, that, at variance with $\mathbf{x}^+$ and $\mathbf{x}^-$ the sign of the components of $\mathbf{w}^+$ and $\mathbf{w}^-$ is not fixed. The ReLU hinge condition can therefore be written as

$$0 = \mathbf{w} \cdot \mathbf{x} + b = (\mathbf{w}^+ + \mathbf{w}^-) \cdot (\mathbf{x}^+ + \mathbf{x}^-) + b$$

Now, exploiting the fact that the two spaces $\mathbb{V}^-$ and $\mathbb{V}^+$ are orthogonal,

$$\mathbf{w} \cdot \mathbf{x} + b = \mathbf{w}^+ \cdot \mathbf{x}^+ + \mathbf{w}^- \cdot \mathbf{x}^- + b$$

We will therefore write the ReLU Hinge as two hinges, one in each subspace $\mathbb{V}^-$ and $\mathbb{V}^+$:

$$\hbar^+ := \mathbf{w}^+ \cdot \mathbf{x}^+ + b^+ = 0 \qquad \hbar^- := \mathbf{w}^- \cdot \mathbf{x}^- + b^- = 0$$

Where $b^+ = b + \mathbf{w}^- \cdot \mathbf{x}^-$ and $b^- = b + \mathbf{w}^+ \cdot \mathbf{x}^+$. Those are the conditions for being in the hinge "seen from each subspace $\mathbb{V}^-$ and $\mathbb{V}^+$", namely as a function of only $\mathbf{x}^-$ and $\mathbf{x}^+$ respectively. Now we can write the expansion point for the generalized γ-ule as two separate conditions, one in each subspace $\mathbb{V}^-$ and $\mathbb{V}^+$.

$$\mathbb{V}^+ : \quad \mathbf{x}^+_\alpha \in \hbar^+ \cap \ell^+_{\alpha,\gamma}$$

$$\mathbb{V}^- : \quad \mathbf{x}^-_\alpha \in \hbar^- \cap \ell^-_{\alpha,\gamma}$$

With, eventually, $\mathbf{x}^* = \mathbf{x}^+ + \mathbf{x}^-$

Using this expansion point produces the equivalent of two separate γ-Rules on different subspaces of positive and negative activation. Opportune change of sign that antisymmetrize the weights action and the input component can be introduced for the case of negative activations. Throughout the entire analysis presented in this work, we set $\gamma = 0.3$ for the non-linear layers and $\gamma = 0.0$ for the linear layers. This generalized γ-rule is the one that produces the LRP rule across different neurons, therefore across different Greek indexes, in other words, the value of $q_{\alpha\beta}$ in Eq. (6) of the main text.

S6.2 Relevance as Gradient Computation

Instead of computing the relevance for each neuron separately, we can rewrite the relevance propagation as a gradient propagation (as described in [13]). For a general derivation, let us consider the general first-order propagation rule

$$R_\alpha = \sum_\beta \frac{q_{\alpha\beta}}{\sum_\alpha q_{\alpha\beta}} R_\beta \tag{18}$$

from Equation (6) in the main text, omitting the layer index superscript. The general expression of the contribution from neuron α to neuron β reads $q_{\alpha\beta} = x_\alpha \rho(w_{\alpha\beta})$, where x_α denotes the activation of neuron α, and ρ may represent any modification of the model weights associated with a certain LRP

rule. Plugging this into Equation (18) yields

$$R_\alpha = \sum_\beta \frac{x_\alpha \rho(w_{\alpha\beta})}{\sum_{\alpha,0} x_\alpha \rho(w_{\alpha\beta})} R_\beta \ . \tag{19}$$

Assuming that the neural network can be represented by nested piecewise linear functions we can specify the relevance model

$$R_\alpha := x_\alpha c_\alpha \tag{20}$$

at each layer. This yields

$$c_\alpha = \sum_\beta \frac{x_\beta \rho(w_{\alpha\beta})}{\sum_{\alpha,0} x_\alpha \rho(w_{\alpha\beta})} c_\beta \ . \tag{21}$$

With the modified forward pass defined as $z_\beta := \sum_{\alpha,0} x_\alpha \rho\,(w_{\alpha\beta})$, Equation (21) can be written as a gradient computation

$$c_\alpha = \sum_\beta \frac{\partial z_\beta}{\partial x_\alpha} \frac{x_\beta}{z_\beta} c_\beta \ . \tag{22}$$

This allows for computing the relevance attributions via efficient backpropagation, and it generalizes to different LRP-rules [13]. To determine relevance attributions in the input domain, one simply needs to implement the weight modification ρ where necessary and then perform a single backpropagation. This process yields the modified gradient c_α with respect to the input feature x_α. According to our relevance model (cf. Equation (20)), the relevance score of each input neuron is then directly given by the product of these two values.

To derive an expression for the gradient propagation for GNN-LRP, let us consider the aggregation and combine step of the GNN separately. The gradient propagation for the combine step is described by Equation (22). The higher-order nature of the relevance walks actually arises from the aggregation step. The expression for the relevance propagation in the aggregation step is given in Equation (7) in the main text. It reads

$$R_\alpha^{ij} = \frac{\lambda_\alpha^{ij}\ x_\alpha^i}{\sum_i \lambda_\alpha^{ij}\ x_\alpha^i} R_\alpha^j \ , \tag{23}$$

where λ_α^{ij} denotes the edge feature between neuron α on graph node i and neuron α on node j, and x_α^i denotes an entry of the node embedding $\mathbf{x}_\alpha^i$. Choosing the relevance model $R_\alpha^{ij} = x_\alpha^i c_\alpha^{ij}$, we obtain

$$c_\alpha^{ij} = \frac{\lambda_\alpha^{ij}\ x_\alpha^j}{\sum_i \lambda_\alpha^{ij}\ x_\alpha^i} c_\alpha^j \ . \tag{24}$$

And again, we can formulate this as a gradient computation

$$c_\alpha^{ij} = \frac{\partial z_\alpha^j}{\partial x_\alpha^i} \frac{x_\alpha^j}{z_\alpha^j} c_\alpha^j \ . \tag{25}$$

with $z_\alpha^j = \sum_i \lambda_\alpha^{ij} x_\alpha^i$ denoting the forward pass.

The crucial difference to Equation (22) is that now we do not sum over all proceeding nodes. This way in each aggregation step we span new relevance branches associated with the relevance walks (cf. Fig. 1 in the main text). In practice, this can be implemented by masking all nodes not directly involved in the walk of interest. For more details regarding the masking procedure please refer to [14].

As mentioned in the main text in Section 2.2, what makes SO3Net and PaiNN stand out against SchNet are their SO3-equivariant features with rotation order $l_{\max} > 0$. This means that SO3Net and PaiNN contain scalar feature representations ($l = 0$) and in addition to that also equivariant features of higher rotation order up to $l_{\max}$. Equivalent to SchNet, the scalar features ($l = 0$) of PaiNN and SO3Net are embedded based on the respective atomic numbers. The equivariant features associated with $l > 0$ are initially embedded as 0-vectors. During the message passing, the equivariant features will be mixed with scalar features, and thus, influence the resulting scalar feature representation.

The models utilized in this work have the energy as an output. The forces are calculated from the first derivative of the model output with respect to the atomic positions. Since the energy is invariant with respect to rotations, the energy is predicted by a forward pass through a multilayer perceptron solely based on the invariant (scalar) features. Equivalently to previous explanations for SchNet [14, 15], we explain the predictions of PaiNN and SO3Net with respect to their scalar atomic features ($l = 0$). The reasoning for this is on the one side that the energy is predicted from the scalar features and on the other side that equivariant features are embedded as 0-vectors which would result in zero relevance attributions.

Data Availability

Simulation data and scripts to reproduce the analysis and the plots shown in the manuscript are accessible on zenodo under https://zenodo.org/records/17068397. The codebase is available at https://github.com/jnsLs/gnn-lrp-cg.git [16].

References

[1] Schütt, K. T., Sauceda, H. E., Kindermans, P.-J., Tkatchenko, A. & Müller, K.-R. SchNet - a deep learning architecture for molecules and materials. *J. Chem. Phys.* **148**, 241722 (2018).

[2] Monroe, J. I. & Shell, M. S. Decoding signatures of structure, bulk thermodynamics, and solvation in three-body angle distributions of rigid water models. *J. Chem. Phys.* **151**, 094501 (2019).

[3] Stock, P. *et al.* Unraveling hydrophobic interactions at the molecular scale using force spectroscopy and molecular dynamics simulations. *ACS Nano* **11**, 2586–2597 (2017).

[4] Chau, P.-L. & Hardwick, A. J. A new order parameter for tetrahedral configurations. *Mol. Phys.* **93**, 511–518 (1998).

[5] Errington, J. R. & Debenedetti, P. G. Relationship between structural order and the anomalies of liquid water. *Nature* **409**, 318–321 (2001).

[6] Duboué-Dijon, E. & Laage, D. Characterization of the local structure in liquid water by various order parameters. *J. Phys. Chem. B* **119**, 8406–8418 (2015).

[7] Alon, U. & Yahav, E. On the bottleneck of graph neural networks and its practical implications. *arXiv preprint arXiv:2006.05205* (2020).

[8] Hankins, D., Moskowitz, J. W. & Stillinger, F. H. Water molecule interactions. *J. Chem. Phys.* **53**, 4544–4554 (1970). URL https://doi.org/10.1063/1.1673986.

[9] Medders, G. R., Götz, A. W., Morales, M. A., Bajaj, P. & Paesani, F. On the representation of many-body interactions in water. *J. Chem. Phys.* **143**, 104102 (2015).

[10] Reddy, S. K. *et al.* On the accuracy of the MB-pol many-body potential for water: Interaction energies, vibrational frequencies, and classical thermodynamic and dynamical properties from clusters to liquid water and ice. *J. Chem. Phys.* **145**, 194504 (2016). URL https://doi.org/10.1063/1.4967719.

[11] Zhai, Y., Caruso, A., Bore, S. L., Luo, Z. & Paesani, F. A "short blanket" dilemma for a state-of-the-art neural network potential for water: Reproducing experimental properties or the physics of the underlying many-body interactions? *J. Chem. Phys.* **158**, 084111 (2023).

[12] Montavon, G., Samek, W. & Müller, K.-R. Methods for interpreting and understanding deep neural networks. *Digit. Signal Process.* **73**, 1–15 (2018).

[13] Montavon, G., Binder, A., Lapuschkin, S., Samek, W. & Müller, K.-R. Layer-wise relevance propagation: An overview. In Samek, W., Montavon, G., Vedaldi, A., Hansen, L. K. & Müller, K.-R. (eds.) *Explainable AI: Interpreting, Explaining and Visualizing Deep Learning*, 193–209 (Springer International Publishing, 2019).

[14] Schnake, T. *et al.* Higher-order explanations of graph neural networks via relevant walks. *IEEE Trans Pattern Anal Mach Intell* **44**, 7581–7596 (2022).

[15] Letzgus, S. *et al.* Toward explainable artificial intelligence for regression models: A methodological

perspective. *IEEE Signal Processing Magazine* **39**, 40–58 (2022).

[16] Bonneau, K. & Lederer, J. jnsls/gnn-lrp-cg: 1.0.0 (2025). URL https://doi.org/10.5281/zenodo.17087871.
